# Cognitive effort-avoidance in patients with schizophrenia can reflect Amotivation: an event-related potential study

**DOI:** 10.1186/s12888-020-02744-4

**Published:** 2020-07-01

**Authors:** Y. X. Lin, Li Jun Zhang, Liang Ying, Qiang Zhou

**Affiliations:** 1 Department of Psychology, Wenzhou Medical University, Chashan University Town, Chashan, Wenzhou, 325035 Zhejiang Province China; 2 Psychiatric Rehabilitation, Wenzhou Seventh Hospital, Wenzhou, China; 3 Wenzhou Medical University, Wenzhou, China

**Keywords:** Schizophrenia, Amotivation, Cognitive effort-avoidance, Demand selection task, Contingent negative variation (CNV)

## Abstract

**Background:**

Amotivation is regarded as a core negative symptom in patients with schizophrenia. There are currently no objective methods for assessing and measuring amotivation in the scientific literature, only a trend towards assessing motivation using effort-orientated, decision-making tasks. However, it remains inconclusive as to whether cognitive effort-avoidance in patients with schizophrenia can reflect their amotivation. Therefore, this study aimed to find out whether cognitive effort-avoidance in patients with schizophrenia can reflect their amotivation.

**Methods:**

In total, 28 patients with schizophrenia and 27 healthy controls were selected as participants. The demand selection task (DST) was adapted according to the feedback-based Guilty Knowledge Test (GKT) delayed response paradigm, which was combined with the mean amplitude of contingent negative variation (CNV), considered as the criterion of motivation.

**Results:**

Our results showed that: (1) patients with schizophrenia showed a lower CNV amplitude for the target stimuli compared to the probe stimuli, whereas the control group showed the opposite trend (*P* < 0.05); (2) among patients with schizophrenia, the high cognitive effort-avoidance group showed a smaller CNV amplitude for the target stimuli compared to the probe stimuli, whereas the low cognitive effort avoidance group showed a higher CNV amplitude for the target stimuli compared to the probe stimuli; the opposite trend was observed in the control group (*P* < 0.05).

**Conclusion:**

These findings support the claim that CNV amplitude can be used as a criterion for detecting amotivation in patients with schizophrenia. Within the context of the DST, the high and low cognitive effort-avoidance of patients with schizophrenia can reflect their state of amotivation; patients with high cognitive effort-avoidance showed severe amotivation.

## Background

Negative symptoms—mainly characterized by emotional, information processing and behavioural deficits—are core constituents of schizophrenia [12]. Schizophrenia patients can present a variety of behavioural and motivational deficits [28], and some researchers have suggested that amotivation is the central negative symptom [13]. In recent years, researchers have begun to apply effort-orientated, decision-making tasks in their assessments of symptoms, especially in their assessments of amotivation [11, 17, 38]. However, further research is still needed to verify whether the avoidance of cognitive effort in patients can reflect their motive state.

Previous studies have mostly used scale assessments as the criterion for amotivation, which are limited by their dual lack of objectivity and accuracy. As event-related potentials (ERPs) are highly correlated with patient condition [3, 4, 10, 33], they provide a more reliable means for describing the characteristics of patients’ symptoms. Gerrig and Zimbardo (2013) proposed that motivation is the process by which an individual initiate, directs and maintains their physical or mental activities. In other words, motivation is the individual’s mental state while they are performing a task; which means that the measurement of motivation will require a relatively high temporal resolution. Therefore, ERPs, which have a high temporal resolution, provide us with the possibility of objectively quantifying and revealing amotivation.

In this study, we propose that the inconsistencies among the results of previous studies may be related to the types of tasks adopted in cognitive effort decision-making [11] also points out that the discrepancies in experimental findings may have resulted from the differences in effort-based tasks. Furthermore, it has been shown that deceptive responses consume greater cognitive resources [1]. Therefore, by building on the existing demand selection task (DST) paradigm, this study introduces a new cognitive task—the deception task.

The introduction of the mental processes of deception means that we will need a corresponding lie-detection paradigm. In this regard, the Guilty Knowledge Test (GKT) paradigm has been widely used in lie-detection studies [19]. Using the GKT delayed response task, Cui [9] found that target stimuli evoked the largest contingent negative variation (CNV) without feedback, whereas probe stimuli evoked the largest CNV with feedback; therefore, supporting the differentiation in CNV, under the influence of response motivation. In summary, this study aims to (1) incorporate the target and probe stimuli from the feedback-based GKT delayed response paradigm into the DST paradigm, (2) use the selection rates of high-effort option (see DST task below) to group the participants into high and low cognitive effort-avoidance groups based on the criterion of *M* ± 0.67*SD* [5, 25, 31] which may shape the characteristics of both two groups more remarkably. Specifically, as the indicator is the selection rates of high-effort option, if the participant’s counterpart is 0.67 standard deviation greater/smaller than the average, then we would group him into low/high cognitive effort-avoidance group. and (3) employ CNV as the quantitative indicator of motivation: all in order to explore whether the cognitive effort-avoidance of patients with schizophrenia can reflect their amotivation.

Due to the presence of abnormalities in the emotional regulation of patients with schizophrenia [8, 21, 34], this study evoked deceptive behaviours by requiring the participants to assist the researcher in the testing of a lie detector—thereby avoiding the adverse effects of ‘scenario simulation’ on patients. One PET study found that there were differences in the cognitive processes of spontaneous and passive deception [2]. As such, spontaneity may affect CNV amplitude. In light of this, we propose the following hypotheses: (1) CNV can be used to detect amotivation in patients with schizophrenia, which will manifest as greater CNV amplitudes for target stimuli than probe stimuli in the control group, with the opposite trend manifesting in the patient group; and (2) the selection of high or low cognitive effort-avoidance by patients with schizophrenia in the DST will reflect their amotivation state, whereby patients with high cognitive effort-avoidance will show severe amotivation and have smaller CNV amplitudes for target stimuli than probe stimuli; and patients with low cognitive effort-avoidance will not show amotivation, having the opposite trend in CNV dissociation.

## Method

### Participants

Outpatients and inpatients from the psychiatric department of the Seventh People’s Hospital of Wenzhou, with schizophrenia diagnoses, were randomly selected as participants. Participants must: (1) meet the DSM-IV diagnostic criteria for schizophrenia; (2) not have organic mental disorders, or mental disorders caused by psychoactive or non-addictive substances; (3) be taking a stable dose of drugs for at least 4 weeks during the course of the study; (4) be literate and educated above the primary school level; (5) be right-handed; and (6) score above 35 points on the Brief Psychiatric Rating Scale (BPRS). Members of the control group must: (1) not have a history of mental disorders, neurological disorders or serious physical illness, not have a family history of mental disorders, and not be taking psychotropic drugs; and (2) be matched in terms of gender(*χ*^2^ = 0.093, *P* = 0.760), age(*t* (53) =0.547, *P* = 0.587, *d* = 0.150), education level(*t* (53) =0.487, *P* = 0.628, *d* = 0.134), handedness (all are right-hander) and other indicators as far as possible.

The normal participants signed consent forms and participated voluntarily in the study and consent from guardians was obtained from guardians of schizophrenia participants. None had previously participated in similar experiments. The participants were given the appropriate reimbursement after the experiment. See Table 1 for more details.

**Table 1:** Participant demographics

Group	***N***	M/F	Age	Education	BRPS total	Course of disease
**SC**	**28**	**23/5**	**38.893 (8.439)**	**9.786 (2.250)**	**44.500 (3.697)**	**12.857 (5.290)**
**HC**	**27**	**23/4**	**37.111 (14.939)**	**10.19 (3.690)**		

*SC* people with schizophrenia, *HC* healthy controls, *M* males, *F* female

Standard deviation in parentheses

### Stimuli

The cognitive effort decision-making materials included a picture showing a pair of circles with different colours (#1 and #2)—only one of which could be selected at a time. After making a selection, a blue or yellow word would appear inside the circle. If the word was blue, the participants were asked to determine whether they had seen it before; if the word was yellow, they were asked to determine if the number of characters was equal to 3. When a circle of one colour was selected, there was a 90% probability that the colour of the presented word would be the same colour as the word presented in the previous selection. This is known as the low-effort “spot.” If the circle of the other colour was selected, there was a 10% probability that the colour of the presented word would be the same colour as the word presented in the previous selection. This is known as the high-effort “spot.”

The lie-detection materials were based on the contents of the simulated murder questionnaire [9], where a word was selected from among the four categories of names, time, murder weapon and body bag; which the participant was asked to memorize before the experiment. Then, another four words were selected from the four respective categories, which were not revealed to the participant before the lie-detection test. From the time the participant entered the laboratory to the time of the lie detection test, the memorized word was regarded as the word the participant had seen, while the non-memorized words were regarded as words the participant had not seen.

### Procedure

Before the start of the experiment, the participants were informed that there was a lie detector that needed to be tested. They were then asked to help with testing the lie detector, and were told they would be reimbursed at the end of the experiment.

**Fig. 1:**
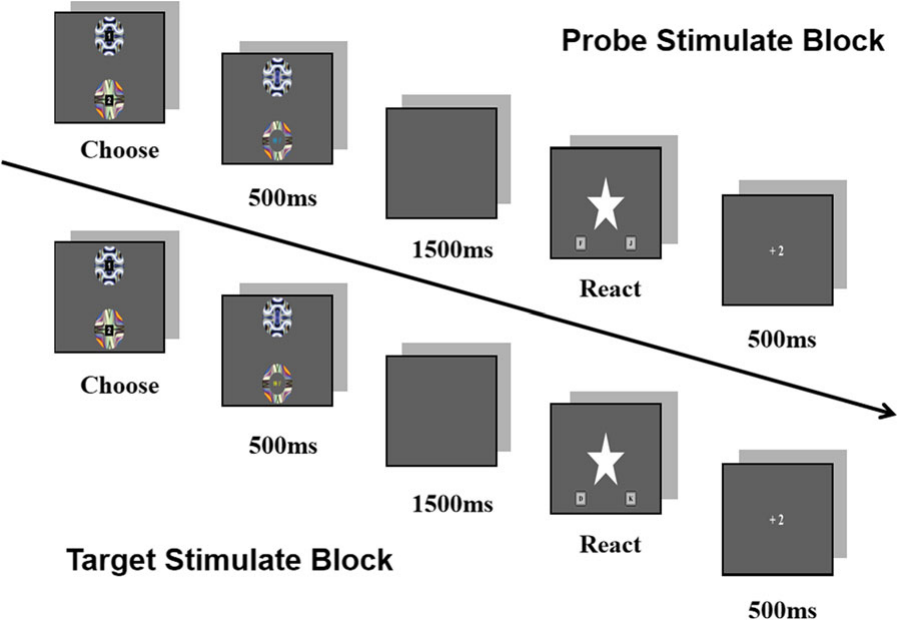
Flow Diagram of Experiment. (First, a pair of circles appeared on the screen with different colors (#1 and #2). After the subject pressed the corresponding key to select, the stimulus appeared in the selected circle for 500 ms. The subject needed to recognize the stimulus but not to react. A black screen after the stimulus disappeared was given for the subject to prepare for reaction. After that, the prompt “☆” appeared, and the duration is not limited. When seeing the prompt, the subject can answer whether he has seen it or whether the word is less than or equal to 3. Then a feedback (“ +2 ”or“ -2 ”) would be given. The feedback was preset, including 50% of “+2” and 50% of “-2”. The two kinds of feedback appeared randomly. The feedback lasted for 500 ms, then the subject would go to next trail)

During the experiment, the participants sat in a quiet laboratory, with both eyes fixated on the centre of the screen at a distance of approximately 60 cm. The participants were asked to minimize their movements; to avoid blinking between the stimulus onset and the key press; while also responding as quickly and accurately as possible. There were 40 selections per block, and there were 8 blocks. The participants could take a break after every 4 blocks. The experimental flow is shown in Fig. 1.

### ERP data recording and analysis

A 64-channel EEG system (ANT Neuroscan, the Netherlands) was used to record EEG. The electrode AFz was selected as grounding. All electrode impedances were maintained below 10 kΩ. The leads of both mastoids were used as the reference electrodes. The sampling rate was 500 Hz/channel. The data was processed offline with ASA1.0 and artifacts were fully excluded. The filter bandpass off-line was 0.05—80 Hz. Amplitudes greater than ±80 μV were regarded as anomalies, and were automatically rejected. We also marked eye movement artifacts in ASA1.0 and then removed them using principal component analysis (PCA) provided by ASA1.0. The ERP responses between the stimulus onset and the key press were analysed for the two groups. The ERP components after the stimulus onset were examined and the analysed epoch was 2000 ms after the stimulus onset. The baseline was 200 ms before the stimulus onset. Mean amplitude for the CNV served as the main dependent variable. The programming and collection of behavioural data were performed using E-Prime 2.0; statistical analyses were performed using SPSS 22.0 including repeated measures ANOVA, T test, the Mauchly sphericity test and Least Significant Difference (LSD) test. Moreover, Greenhouse Geisser test,if necessary, would be used to correct for non-homogenous values. We used 0.05 as the critical significance level.

## Results

### Behavioural data

The participants were divided into the high and low cognitive effort-avoidance groups, based on their selection rate for the high-effort “spot” (*M* ± 0.67*SD*). A two-way (patient condition * cognitive effort-avoidance) analysis of variance (ANOVA) was performed on the selection rate of high-effort option, which showed that the main effect of cognitive effort-avoidance was significant (high cognitive effort-avoidance < low cognitive effort-avoidance), *F* (1,16) = 182.352, *P* = 0.000, *η*^*2*^ = 0.919, the main effect of patient condition was not significant, *F* (1,16) =0.484, *P* = 0.497, *η*^*2*^ = 0.029, and the interaction effect between these two factors was not significant, *F* (1,16) =0.001, *P* = 0.973, *η*^*2*^ = 0.000.

A three-way mixed design repeated measures; ANOVA was performed on the accuracy rate and reaction time (cognitive effort-avoidance * stimulus type * patient condition). The results of analysing the accuracy rates showed that: (1) the main effect of cognitive effort-avoidance was significant, *F* (1,16) = 5.758, *P =* 0.029, *η*^*2*^ *=* 0.265; and (2) the interaction effect between patient condition and cognitive effort-avoidance was marginally significant, *F* (1,16) = 4.120, *P* = 0.059, *η*^*2*^ *=* 0.205. Simple effects analysis indicated that, in the patient group, the difference in the accuracy rates between the high and low cognitive effort groups was not significant(*F* (1,9) = 0.086, *P* = 0.775); in the control group, the accuracy rate of the high cognitive effort avoidance group was significantly lower than that of the low cognitive effort-avoidance group(*F* (1,7) = 7.716, *P* = 0.027). Analysis of the reaction times showed that: (1) the main effect of stimulus type was significant, *F* (1,16) = 4.655, *P =* 0.047, *η*^*2*^ *=* 0.225; the reaction time for the target stimuli was significantly higher than that for the probe stimuli, and (2) the interaction effect between stimulus type and cognitive effort-avoidance was marginally significant, *F* (1,16) = 3.849, *P* = 0.067, *η*^2^ = 0.194. Simple effects analysis indicated that, in the high cognitive effort groups, the difference in the reaction times between the probe stimuli and target stimuli was marginally significant(*F* (1,8) = 4.091, *P* = 0.078); in the low cognitive effort groups, the difference in the reaction times between the probe stimuli and target stimuli was not significant(*F* (1,10) = 0.122, *P* = 0.734) and (3) the interaction effect between patient condition and cognitive effort-avoidance was marginally significant, *F* (1,16) = 3.820, *P* = 0.068, *η*^*2*^ *=* 0.193. Simple effects analysis indicated that, in the patient group, the difference in the reaction times between the high and low cognitive effort groups was not significant(*F* (1,9) = 0.567, *P* = 0.471); in the control group, the reaction times of the high cognitive effort avoidance group was significantly higher than that of the low cognitive effort-avoidance group(*F* (1,7) = 6.628, *P* = 0.034). No other effects of interest were significant. See Table 2 and Table 3 for details.

**Table 2:** Behavior date demonstration

		SC				HC			
		HCA(32.313%)		LCA(60.000%)		HCA(33.672%)		LCA(61.500%)	
		Probe	Target	Probe	Target	Probe	Target	Probe	Target
**Accuracy (%)**	***M***	**0.794**	**0.838**	**0.853**	**0.827**	**0.660**	**0.603**	**0.885**	**0.940**
	***SD***	**0.177**	**0.101**	**0.139**	**0.140**	**0.263**	**0.162**	**0.201**	**0.012**
**RT (ms)**	***M***	**1027.804**	**1285.726**	**1410.617**	**1576.199**	**973.898**	**1620.545**	**586.894**	**464.288**
	***SD***	**563.82**	**924.379**	**628.910**	**880.271**	**510.378**	**1003.258**	**126.990**	**143.280**

*HCA* high cognitive avoidance, *LCA* low cognitive avoidance, *RT* react time

High effort patch selection rate in parentheses

**Table 3:** Behavioral statistical results demonstration

Factors	Accuracy			Reaction Times		
	*F*	*P*	*η*^*2*^	*F*	*P*	*η*^*2*^
stimulus type	0.012	0.915	0.001	4.655	0.047	0.225
patient condition	0.767	0.394	0.046	2.129	0.164	0.117
cognitive effort-avoidance	5.758	0.029	0.265	0.588	0.454	0.035
stimulus type✖patient condition	0.020	0.889	0.001	0.052	0.822	0.03
stimulus type✖cognitive effort-avoidance	0.093	0.765	0.006	3.849	0.067	0.194
patient condition✖cognitive effort-avoidance	4.120	0.059	0.205	3.820	0.068	0.193
patient condition✖stimulus type✖cognitive effort-avoidance	1.821	0.196	0.102	2.376	0.143	0.129

### ERP analysis

#### CNV dissociation

The grand average map revealed that CNV was evoked between the stimulus onset and the key press. A total of 14 electrode sites (Fz, FCz, Cz, Pz, F3, F4, FC3, FC4, C3, C4, CP3, CP4, P3 and P4) were selected for this study. The CNVs evoked by different stimuli were analysed in each group. One patient was excluded from the EEG analysis due to excessive eye movement artifacts.

**Fig. 2:**
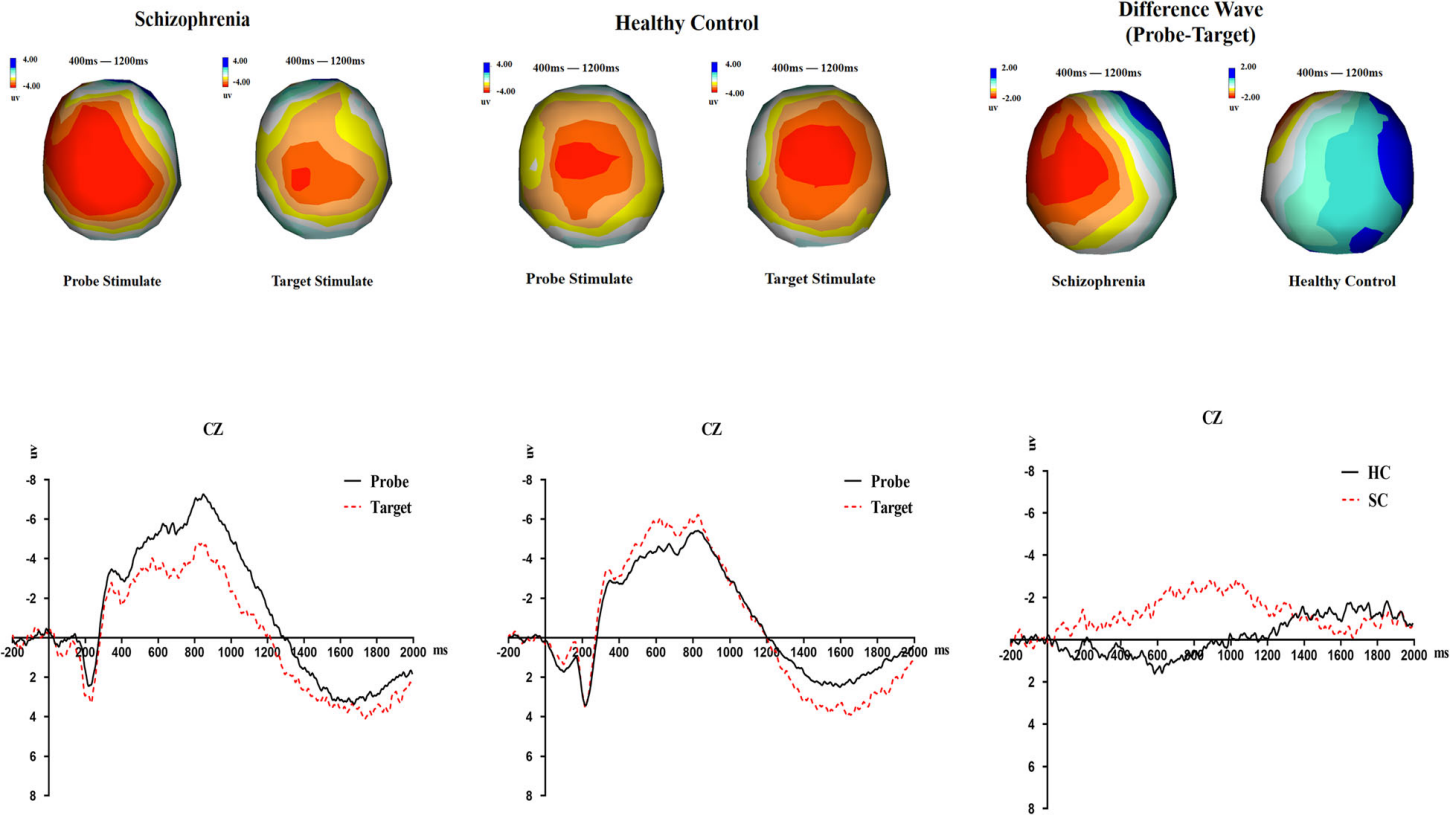
ERPs and Topographical map of brain for all conditions at CZ cite

We noted that Jang et al. [24] have once studied CNV around 400 ms which is a little similar to our study and Cui et al. [9] have discussed early stage CNV in simple GKT task by selecting the length of time window about 800 ms, which was thought more related to motivation. Thus, a three-way mixed design repeated measures ANOVA was performed using the mean CNV amplitude (from 400 ms to 1200 ms) as the indicator. The results indicated that: (1) the main effect of electrode sites was significant, *F* (13,676) = 3.318, *P* = 0.000, *η*^*2*^ = 0.060; (2) the interaction effect between electrode sites and stimulus types was significant, *F* (13,676) = 5.252, *P* = 0.000, *η*^*2*^ = 0.092; (3) the interaction effect between electrode sites and patient condition was marginally significant, *F* (13,676) = 1.580, *P* = 0.086, *η*^*2*^ = 0.029; (4) the interaction effect between stimulus types and patient condition was marginally significant, *F* (13,52) = 3.474, *P* = 0.068, *η*^*2*^ = 0.063;and (5) the three-way interaction effect was significant, *F* (13,676) = 1.830, *P* = 0.035, *η*^*2*^ = 0.034. Simple simple effects analysis showed that: (1) in the patient group, according to the Least Significant Difference (LSD) test,the probe stimuli evoked the largest CNV at Cz, which was not significantly different from those at FCz(*P* = 0.106) and C3(*P* = 0,271), but significantly higher than those at the remaining electrodes(F3:*P* = 0.021; F4:*P* = 0.000; Fz:*P* = 0.002; FC3:*P* = 0.045; FC4:*P* = 0.008; C4:*P* = 0.001; CP4:*P* = 0.025; CP3:*P* = 0.013; P3:*P* = 0.03; Pz:*P* = 0.016; P4:*P* = 0.005); and (2) the target stimuli evoked the largest CNV at Cz, which was significantly higher than those at F3(*P* = 0.03), F4(*P* = 0.025), FZ(*P* = 0.036), FC3(*P* = 0.023), CP3(*P* = 0.006) and P3 (*P* = 0.003), but not significantly different from those of the remaining electrodes (FCz:*P* = 0.267; FC4:*P* = 0.092; C3:*P* = 0.390; C4:*P* = 0.197; CP4:*P* = 0.618; Pz:*P* = 0.458; P4:*P* = 0.235). The CNV amplitudes evoked by the probe and target stimuli were significantly or marginally significantly different at F3(*P* = 0.044), Fz(*P* = 0.098), FC3(*P* = 0.041), FCz(*P* = 0.055), C3(*P* = 0.019), Cz(*P* = 0.046) and CP3(*P* = 0.031); the difference was not significant for the remaining electrode sites(F4:*P* = 0.128; FC4:*P* = 0.939; C4:*P* = 0.548; CP4:*P* = 0.619; P3:*P* = 0.122; Pz:*P* = 0.296; P4:*P* = 0.905). In the control group, according to the Least Significant Difference (LSD) test, the probe stimuli evoked the largest CNV at Cz, which was not significantly different from those at F3(*P* = 0.153), Fz(*P* = 0.100), P3(*P* = 0.105) and Pz(*P* = 0.113), but significantly or marginally significantly higher than those at the remaining electrodes(F4:*P* = 0.004; FC3:*P* = 0.002; FCz:*P* = 0.067; FC4:*P* = 0.012; C3:*P* = 0.053; C4:*P* = 0.015; CP4:*P* = 0.046; CP3:*P* = 0.088; P4:*P* = 0.039); while the target stimuli evoked the largest CNV at Cz, which was marginally significantly different from those at FCz(*P* = 0.066), FC4(*P* = 0.095), C4*(P* = 0.055), CP4(*P* = 0.082) and Pz(*P* = 0.068), and significantly higher than those of the remaining electrodes(F3:*P* = 0.003; F4:*P* = 0.010; Fz:*P* = 0.017; FC3:*P* = 0.000; C3:*P* = 0.041; CP3:*P* = 0.022; P3:*P* = 0.045; P4:*P* = 0.044). The CNV amplitudes evoked by the probe and target stimuli were significantly different at F3(*P* = 0.018), FC4(*P* = 0.001), C4(*P* = 0.001) and CP4(*P* = 0.019); the difference was not significant for the remaining electrode sites(F4:*P* = 0.135; Fz:*P* = 0.820; FC3:*P* = 0.135; FCz:*P* = 0.137; C3:*P* = 0.788; Cz:*P* = 0.174; CP3:*P* = 0.919; P3:*P* = 0.956; Pz:*P* = 0.799; P4:*P* = 0.453). We selected Cz, which had the largest CNV amplitude, and plotted the topographic maps and waveforms of different stimuli for each group. The independent *T* test revealed that the waveforms at Cz were significantly different between the two groups, *t* (52) = − 2.372, *P* = 0.021, *d* = 0.658. No other effects of interest were significant. See Table 4 and Fig. 2.

**Table 4:** EEG statistical results demonstration

Factors	CNV mean amplitude		
	*F*	*P*	*η*^*2*^
electrode sites (in CNV Dissociation)	3.318	0.000	0.060
stimulus types (in CNV Dissociation)	1.254	0.268	0.024
patient condition (in CNV Dissociation)	0.037	0.849	0.001
electrode sites✖stimulus types (in CNV Dissociation)	5.252	0.000	0.092
electrode sites✖patient condition (in CNV Dissociation)	1.580	0.086	0.029
stimulus types✖patient condition (in CNV Dissociation)	3.474	0.068	0.063
electrode sites✖stimulus types✖patient condition (in CNV Dissociation)	1.830	0.035	0.034
stimulus type (in Comparison of Amotivation)	0.285	0.601	0.017
patient condition (in Comparison of Amotivation)	0.091	0.767	0.006
cognitive effort-avoidance (in Comparison of Amotivation)	0.756	0.397	0.045
stimulus type✖patient condition (in Comparison of Amotivation)	0.432	0.521	0.026
stimulus type✖cognitive effort-avoidance (in Comparison of Amotivation)	0.005	0.942	0.000
patient condition✖cognitive effort-avoidance (in Comparison of Amotivation)	4.929	0.041	0.236
patient condition✖stimulus type✖cognitive effort-avoidance (in Comparison of Amotivation)	4.984	0.040	0.238

#### Comparison of Amotivation

**Fig. 3:**
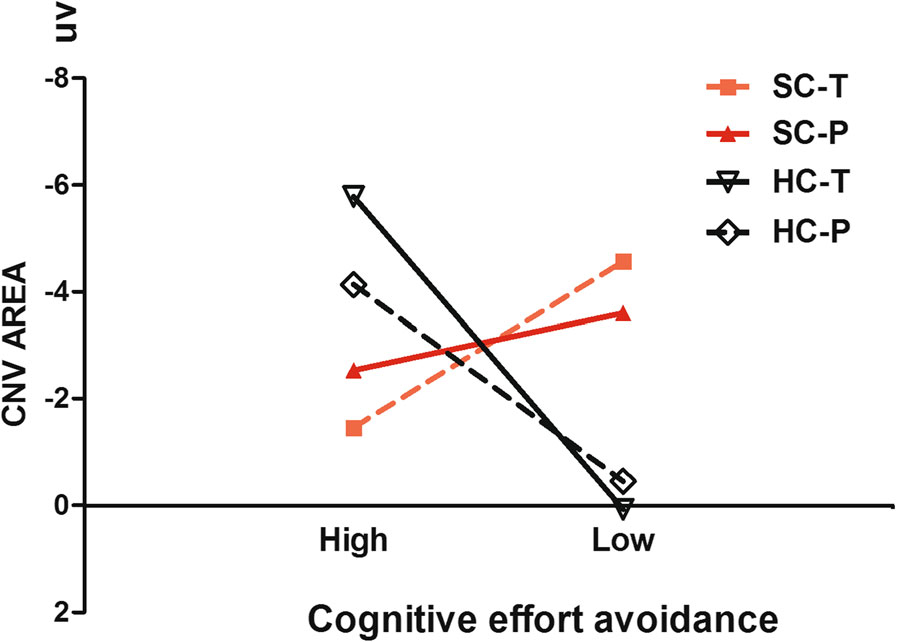
The interaction effect between patient condition and cognitive effort-avoidance in CNV amplitude

A three-way (cognitive effort-avoidance * stimulus type * patient condition) mixed design repeated measures ANOVA was performed using the mean CNV amplitude (from 400 ms to 1200 ms) at Cz as the indicator. The results indicated that: (1) the interaction effect between patient condition and cognitive effort-avoidance was significant, *F* (1,16) =4.929, *P* = 0.041, *η*^*2*^ = 0.236; and (2) the three-way interaction effect was significant, *F* (1,16) =4.984, *P* = 0.040, *η*^*2*^ = 0.238. The remaining effects were not significant (See Table 4). The results of simple simple effect analysis showed that: (1) when presented with the target stimuli, the main effect of cognitive effort-avoidance was not significant in the patient group, *t* (1,9) = 1.348, *P* = 0.211, *d* = 0.899; and (2) the main effect of cognitive effort-avoidance was marginally significant in the control group, *t* (1,7) = − 2.180, *P* = 0.066, *d* = 1.648. When presented with the probe stimuli, the main effect of cognitive effort-avoidance was not significant in the patient group, *t* (1,9) =0.638, *P* = 0.540, *d* = 0.425; while the main effect of cognitive effort-avoidance was not significant in the control group, *t* (1,7) = − 1.469, *P* = 0.185, *d* = 1.110. See Fig. 3.

## Discussion

Previous studies have focused on exploring whether the behavioural performance of patients with schizophrenia can objectively reflect their amotivation. However, the use of clinical scales as the criterion of validity would inevitably go against their intended aim of achieving objectivity. In this study, we consolidated the previous findings showing that CNV can be affected by motivation, and used EEG data as the criterion of validity in order to examine whether cognitive effort-avoidance in patients with schizophrenia can reflect their amotivation.

One of our key findings was that while CNV dissociation was observed when the two groups of participants were presented with two types of tasks, the groups showed opposing trends of dissociation. Hypothesis 1, as proposed in this study, was verified by our findings. The topographic maps indicated that when the patient group was presented with the target stimuli, there was a substantial reduction in their left cortical activity; whereas the opposite trend was observed in the control group. Treadway et al. [39] found that activity in the left striatum and the left ventromedial prefrontal cortex were correlated with a willingness to expend effort, thus indicating that the difference in CNV dissociation could reflect amotivation. Previous studies have generally found that patients with schizophrenia had deficits in switching [23, 32]. Studies have also shown that patients with schizophrenia have a tendency towards hyper-focusing; which is the tendency to concentrate limited resources on a specific stimulus, while ignoring other stimuli [27, 30, 35]. In addition, Soriano [36] and Westerhausen [42] found that patients with schizophrenia have impaired inhibitory functions, which are a prerequisite for the smooth implementation of other executive functions [29]. When patients were presented with the target stimuli, they may have been unable to suppress the dominant response and displayed an impaired allocation of mental resources—thus resulting in lower cognitive input and motivation, which led to a smaller CNV amplitude compared with the probe stimuli. This finding also indicates that the amotivation of patients with schizophrenia may occur when patients are required to perform multiple tasks, whereas patients do not exhibit amotivation when performing a single task, but may instead show stronger motivation. Therefore, during the rehabilitation of patients’ cognitive functions, it may be beneficial to add targeted dual- or multi-task training. Although, this study could not exclude the effect on motivation brougt by medication since we only asked that patients were in same type and dose. Interestingly, we found the changes of right prefrontal cortex activity in patients when presented with the target stimuli are similar to the control group from differential wave topographic map. Since dopamine receptors are widely distributed throughout the brain and given that the right prefrontal cortex is also thought related to motivation [6], this implies the effect of medication on amotivation may be slight at least in this study and CNV may be mainly influenced by individuals’ willingness to expend effort.

Another key finding of this study was that patients with schizophrenia with different degrees of cognitive effort-avoidance showed opposing trends of CNV dissociation. Given that Hypothesis 1 has been verified, this result supports Hypothesis 2. Gold [16] proposed that the detection of cognitive effort can affect cognitive effort-avoidance. The detection of cognitive effort may be related to the patient’s weighing of costs against benefits. A piece of latent information in the DST is that the participant’s selection will not affect their final reward [16, 26]; hence selecting the high-effort “spot” when completing the experiment would result in a lower cost-to-benefit ratio. Dopamine plays an important role in both cost-benefit assessment and weighing effort costs [13]. Changes in its levels can have an impact on an individual’s degree of expended effort [41] and can regulate their sensitivity to resources [14]. Studies have shown that abnormal striatal dopamine release is one of the mechanisms underlying the pathogenesis of schizophrenia [15, 22]. On top of their insufficient resources, the high cognitive effort-avoidance group was also affected by dopamine abnormalities; hence they were more precise when weighing up resource consumption and costs, and were more sensitive to cognitive effort-detection. Tending towards the low-effort “spot” in decision-making can guarantee the smooth completion of the task, while also helping to avoid the substantial consumption of resources caused by task switching. The dissociation in the low cognitive effort-avoidance group was basically consistent with that of the control group. Their spot selection tended to be random and did not exhibit significant cognitive effort-avoidance.

It is perhaps surprising that, although opposing trends of CNV dissociation were also observed in the control group, in the different degrees of cognitive effort avoidance, these trends differed from those observed in the patient group. This suggests that the decision-making performance of the healthy population in this study may reflect a different mental state to that in the patient population. The high number of trials in the DST may affect the mental state of the participants [40]. Studies have also shown that CNV include participant’s processing and evaluation of stimuli [18]. We can infer that participants may want to complete the experiment as soon as possible, and performing a single task is undoubtedly the easiest and quickest way to achieve this. Since the target stimuli would waste more time, the high avoidance group may have a more negative evaluation of the target stimuli, ultimately leading to an increased CNV amplitude. The completion of the two tasks by the low cognitive effort-avoidance was superior to that by the high avoidance group. As the low avoidance group was more familiar with the rules and their responses were automatic, they did not need to invest significant cognitive resources; hence their “spot” selection also tended to be more randomized. For the control group, the difference in high and low cognitive effort-avoidance in the DST may only reflect the participant’s evaluation and preferences for the “spots”, rather than their motive state.

This study, however, has some limitations. First, we focused on the amotivation of patients with schizophrenia when completing cognitive tasks, but patients also exhibit amotivation when completing tasks that require physical effort [7, 20]. Therefore, when performing such a task, the validity of using a patient’s amotivation as an indicator to evaluate their overall amotivation is still debatable. Second, we could not explore whether smarter people would notice differences in effort demands and often prefer to take the easier way due to the lack of formal assessment of cognition. The previous study found while the total effect of IQ on effort avoidance was significant, but neither the direct effect of IQ nor the indirect effect through mediation was significant by itself in both two group [16]., which suggests the IQ may not play a pivotal role. Thus, we only ensured that the patients did not have severe cognitive deficits such as dementia, lapse of memory and so on by the report from professional clinician. In addition, the interaction effect of patient condition and stimulus type or cognitive effort-avoidance and stimulus type in patient group on accuracy was not significant, which also implied the patient did not have severe cognitive deficits as the DST could be categorized to dual task that is not easy to be performed.

Anyway, our results could enrich the understanding and diagnosis of the amotivation in patients with schizophrenia. It is suggested that only when patients are in dual or multi-tasking in stead of single task, they would be amotivation, which could be observed by CNV measuring. Besides, the behaviors (cognitive effort-avoidance) in DST may help us diagnose the severity of this kind of amotivation quickly and conveniently. However, this research is only an explorative start and more future work remains to be conducted. (1) To our knowledge, this study is the first one adopted the criterion of *M* ± 0.67*SD* in DST. But there still are many ways to group high and low and most of them are comparative. Therefore, more relative and large sample researches should be conducted to identify which classification method is the best or whether there is a standard line. (2) Same as previous studies [16], the correlation coefficient between the findings and BPRS scores are not significant and low(*r* < 0.2). Does the inconsistency mean low clinical value? However, as mentioned above, the amotivation is measured in dual task that is different from the stage where the patients are in .scale evaluation Thus, is it possible that low correlation coefficient imply the oppositely high necessity of the DST in clinical evaluation as it show us a diverse dimension of state of patients? Long-term follow-up study may tell the clinical value of DST. (3) The future work remains to focus on the specific impact on motivation and performance in DST brought by different medication situation, individual basic cognitive ability(e.g. executive attention, cognitive flexibility and working memory) and changes in the strength of brain network connections. (4) we have noted that the causes of schizophrenia are complex, diverse and highly heterogenous [37], therefore, it is also appealing to explore the impact of various causes on these findings.

## Conclusion

From the findings of this study, the following conclusions can be made: (1) CNV can be used to detect amotivation in patients with schizophrenia; and (2) the high and low cognitive effort-avoidance of patients with schizophrenia when completing the DST can reflect their amotivation state, as patients with high cognitive effort-avoidance showed severe amotivation.

## Data Availability

The datasets used and/or analyzed during the current study are available from the corresponding author on reasonable request.

## Abbreviations

DST: The demand selection task
GKT: The feedback-based Guilty Knowledge Test
CNV: Contingent negative variation
ERPs: Event-related potentials
PET: Positron Emission Computed Tomography
DSM-IV: Diagnostic and Statistical Manual of Mental Disorders, Fourth Edition
BPRS: The Brief Psychiatric Rating Scale
EEG: Electroencephalogram
SPSS: Statistical Product and Service Solutions
